# A FEM Free Vibration Analysis of Variable Stiffness Composite Plates through Hierarchical Modeling

**DOI:** 10.3390/ma16134643

**Published:** 2023-06-27

**Authors:** Gaetano Giunta, Domenico Andrea Iannotta, Marco Montemurro

**Affiliations:** 1 Luxembourg Institute of Science and Technology, 5 Avenue des Hauts-Fourneaux, L-4362 Esch-sur-Alzette, Luxembourg; domenico-andrea.iannotta@list.lu; 2 Doctoral School in Science and Engineering (DSSE), University of Luxembourg, 2 Avenue de l’Université, L-4365 Esch-sur-Alzette, Luxembourg; 3 Arts et Métiers Institute of Technology, Université de Bordeaux, CNRS, INRA, Bordeaux INP, HESAM Université, I2M UMR 5295, F-33405 Talence, France; marco.montemurro@ensam.eu

**Keywords:** free vibration analysis, finite element method, variable angle tow plates, Carrera’s unified formulation, Reissner’s mixed variational theorem

## Abstract

Variable Angle Tow (VAT) laminates offer a promising alternative to classical straight-fiber composites in terms of design and performance. However, analyzing these structures can be more complex due to the introduction of new design variables. Carrera’s unified formulation (CUF) has been successful in previous works for buckling, vibrational, and stress analysis of VAT plates. Typically, one-dimensional (1D) and two-dimensional (2D) CUF models are used, with a linear law describing the fiber orientation variation in the main plane of the structure. The objective of this article is to expand the CUF 2D plate finite elements family to perform free vibration analysis of composite laminated plate structures with curvilinear fibers. The primary contribution is the application of Reissner’s mixed variational theorem (RMVT) to a CUF finite element model. The principle of virtual displacements (PVD) and RMVT are both used as variational statements for the study of monolayer and multilayer VAT plate dynamic behavior. The proposed approach is compared to Abaqus three-dimensional (3D) reference solutions, classical theories and literature results to investigate the effectiveness of the developed models. The results demonstrate that mixed theories provide the best approximation of the reference solution in all cases.

## 1. Introduction

Over the last decades, composite structures have gained significant attention across diverse application fields, including aerospace, automotive and construction, due to their unique properties. Due to their high stiffness-to-weight ratio, composites help to build light structures with interesting mechanical properties. Despite this, a common thought is that the potential of fiber-reinforced structures could be better exploited by improving the directional properties through the variation of the fiber angle along the in-plane directions. The choice to keep the fiber orientation constant in each layer is particularly restrictive for geometries that present geometrical discontinuities such as cut-outs. VAT plates are characterized by an in-plane variation of fiber angle, helping to expand the design space of a specific structure. This is particularly useful for optimization problems, where a wider design space can positively affect the search of an optimal solution. For example, in the context of vibrational analyses, the maximization of fundamental frequencies can be improved by using curvilinear fibers. VATs were originally obtained through automated tape placement (ATP) and automated fiber placement (AFP). ATP helps the automated placement of composite material tapes with a specific angle in order to reproduce a desired path. AFP is similar to ATP, since the main difference is related to the width of the material that is laid down: while ATP handles a tape with a width between 75 and 300 mm, AFP involves the placement of the material with a typical width between 3.1 and 12.7 mm. By consequence, AFP allows for better control of fiber angles, achieving a wider design flexibility; see Dirk et al. [1]. However, automated processes show some limitations related to manufacturing defects, such as gaps and overlaps, or constraints such as the minimum steering radius. These aspects can be partially overcome due to new technologies such as additive manufacturing (AM), also known as 3D printing. AM involves the layer-by-layer deposition of materials to create a three-dimensional object. In the case of variable angle tow composites, AM techniques are used to deposit and cure layers of composite materials with varying fiber orientations; see Zhuo et al. [2]. VAT composites have diverse applications ranging from aerospace engineering and wind energy to automotive and construction contexts, offering enhanced structural performance, weight reduction, and tailored properties for improved efficiency and functionality in a wide range of industries. For example, these materials can be employed in the optimization of aircraft wings to enhance structural weight and fuel consumption, as presented in Brooks et al. [3]. In the space context, VATs can be used for the design of liquid oxygen in order to reduce the mass and increase the payload of space launchers, as discussed by Gren et al. [4]. Despite the significant advantages associated with curvilinear fiber composites, these materials have limitations from both manufacturing and design perspectives. The production of VAT composites can be more complex in comparison with traditional laminates, since the material behavior is strongly affected by process-induced defects. Moreover, not all fiber patterns can be realized, because of the technological limitations that characterize their production. The complexity of analysis is one of the main disadvantages of VATs, because a greater number of unknowns must be taken into account and unfeasible fiber patterns could be obtained during the optimization process.

Several methods for the study of VAT mechanical responses are available in the literature. In the following text, a brief review of these approaches is presented, with a particular focus on free vibration analyses. To the best of the authors’ knowledge, the first works that have been presented on the topic are based on the assumption of a constant fiber angle within each element in a finite element method (FEM) solution. Therefore, the continuous variation of fiber direction was approximated in a step-wise discrete way. This approach can be used in commercial FEM software tools that, at the moment, cannot handle continuous fiber variation. Hyer and Charette [5] and Hyer and Lee [6] used this method to improve the VAT tensile strength and buckling response, respectively. One of the main disadvantages of this step-wise approach is that, as the true variation is continuous, the discrete representation of fiber angle variation imposes a further approximation. A p-version FEM based on the third-order shear deformation theory (TSDT) was applied by Akhavan and Ribeiro [7] to preform vibrational analyses. The results showed that fiber variation helps to increase (or decrease) natural frequencies and that thin plates are more affected by this phenomenon if compared with thick ones. Ribeiro and Akhavan [8] used the p-version FEM approach with elements based on the first-order shear deformation theory (FSDT) to perform non-linear vibration analyses. The advantage of the p-version of the FEM is that the accuracy of the approximation is improved by increasing the order of shape functions over the elements. Vibration analyses were performed on VAT plates with a central circular cut-out considering parabolic fibers by Hachemi et al. [9]. Zhao and Kapania [10] investigated the free vibration of prestressed VAT stiffened plates, where plates and stiffeners were modeled separately through Mindlin plate theory and Timoshenko beam theory, respectively. The compatibility conditions at the interface between the plate and stiffeners were satisfied by using a transformation matrix. Honda and Narita [11] used the classical plate theory within the Ritz method in order to evaluate the natural frequencies and vibrational modes. An experimental approach was used in Rodrigues et al. [12] for the free vibration analysis of a plate with free boundary conditions that was subjected to random excitation via an electromagnetic shaker. Subsequently, the results were compared to the ones obtained through FEM, where a four-node isoparametric element based on the Reissner–Mindlin theory was used. Stodieck et al. [13] showed that curvilinear fibers can be useful for improving the aeroelastic response of composite wings. The Rayleigh–Ritz method and classical lamination theory were used to develop a 1D beam model, considering the assumption of null chamber deformation of the wing chord-wise section. The aeroelastic response was computed by introducing quasi-static aerodynamic forces in a model developed for the plate structural analysis. A parametric study showed that by using VATs, it is possible to influence wing response both positively and negatively.

Curvilinear fibers can improve the modal response, as shown in several works. Abdalla et al. [14] used the classical lamination theory in combination with a successive approximation method in order to solve an optimization problem. The results showed that curvilinear fibers increased the optimal fundamental frequency in comparison with straight ones. A similar approach was presented in Blom et al. [15], where the maximization of the first natural frequency considering manufacturing constraints was obtained for VAT conical shells. In Carvalho et al. [16], a genetic algorithm and shell elements based on FSDT were used for maximization of the fundamental frequency. The multi-scale two-level (MS2L) approach helps to split the optimization problem in two parts. The composite is modeled as an equivalent homogeneous anisotropic plate in the first step, which aims to find the ideal distribution of the polar parameters that represent the mechanical design variables. The main goal of a second step is to establish the best stacking sequence in relation to the mechanical property distribution that has been obtained in the first step. The MS2L method was applied by Montemurro and Catapano [17] to VAT plates in order to optimize the buckling response. In order to evaluate the polar parameters, B-spline surfaces were introduced, while manufacturing constraints were considered during the second step. More details about the MS2L approach can be found in Catapano et al. [18], Montemurro and Catapano [19] and Fiordilino et al. [20], where both stiffness and buckling optimization problems were solved.

VAT structures have also been studied by using Carrera’s unified formulation. CUF is a mathematical framework that helps the derivation of different theories, such as classical lamination plate theory, higher-order shear deformation theories, or LW approaches, within a unique formulation; see Carrera [21,22]. The a priori approximation over the thickness (typical of plates’ structural modeling) can also be freely assumed as a generic combination of functions whose number is a free parameter of the formulation. When polynomial functions are used, as in this article, the expansion order along the thickness of the plate is arbitrary in the formulation, and it can be set when performing a specific analysis. This flexibility is beneficial because it helps to tailor the accuracy and computational efficiency of the analysis to the specific requirements of the problem at hand. Carrera et al. [23] used CUF in order to develop a Navier closed-form solution for the static analysis of isotropic plates under several loading conditions. The same approach was used in Carrera and Giunta [24] in order to perform failure analyses on isotropic plates. A further extension of this method was shown in Giunta et al. [25], where a indentation failure analysis of composite sandwich plates was performed. Giunta et al. [26] performed free vibration analyses of composite beams. In Viglietti et al. [27] and Fallahi et al. [28], free vibration and buckling analyses of VATs were performed through the use of a 1D CUF model. Within this framework, shell models were developed as well for VAT cases in order to perform stress analyses; see Sánchez-Majano et al. [29]. In Pagani and Sánchez-Majano [30,31] and Sánchez-Majano et al. [32], manufacturing defects were taken into account by using stochastic techniques. Vescovini and Dozio [33] used the Ritz method within CUF for vibrational and buckling analyses. A generalization of CUF was developed in order to allow for the use of different expansions for every component of the displacement vector. Demasi et al. [34] applied this approach to the study of VAT plates with an ESL model. A further advantage of CUF is that it can be used in combination with different variational statements. An alternative to the classic PVD is represented by the RMVT, where both displacements and transverse out-of-plane stresses are considered as primary variables. RMVT has been widely used within CUF for the study of straight-fiber composite structures. For example, Carrera and Demasi [35,36] developed RMVT-based CUF models to perform the static analysis of straight fiber plates.

The free vibration analysis is an important problem in engineering (see Babaei et al. [37]), and within this context, CUF has been applied to the study of VATs considering PVD as the main variational statement. For this reason, this work aims to extend this framework with the RMVT formulation in order to develop a family of hierarchical plate finite elements. This will help to better predict the natural frequencies of composite plates characterized by curvilinear fibers. Section 2 shows the theoretical derivation for free vibration problems. Section 3 presents the numerical results where three cases are investigated. Analyses are performed that consider a varying side-to-thickness ratio in order to investigate thin and thick plates, and the differences between models are discussed regarding PVD or RMVT statements. The results are compared to reference solutions for validation. Concluding observations and remarks are presented in Section 4.

## 2. Carrera’s Unified Formulation

A plate is a flat body whose material points lie in the Cartesian closed-point subset

**Figure 1:**
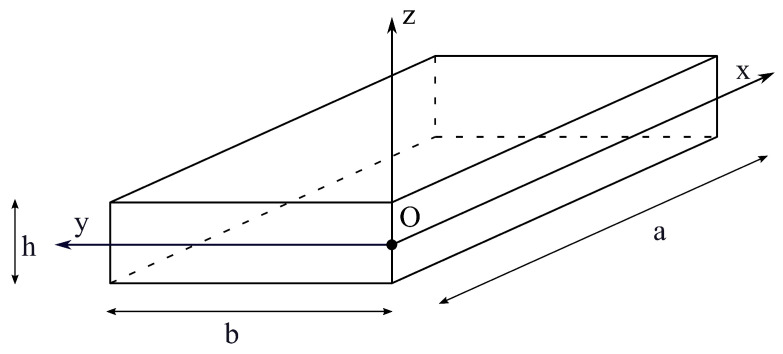
Plate geometry and reference system.

(1) P=Ω×H

of the three-dimensional space $R^{3}$ where:

(2) $$\begin{array}{c} \Omega =\left\{\left(x,y\right)\;:\;\frac{x}{a},\;\frac{y}{b}\in \left[0,1\right]\right\}\subset R^{2},\;\\ H=\left\{z\;:\;\frac{2z}{h}\in \left[-1,1\right]\right\}, \end{array}$$

where *a* and *b* are the dimensions along the two in-plane axes, and *h* measures its thickness along the *z*-axis, where z≪a and *b*. The global reference system and plate geometry are presented in Figure 1.

The displacement field is expressed as:

(3) $$u=\left\{\begin{array}{c} u_{x}\\ u_{y}\\ u_{z} \end{array}\right\}.$$

The strain tensor components can be written in vector form. Two vectors are obtained, representing the in-plane and out-of-plane components:

(4) $$\epsilon_{p}=\left\{\begin{array}{c} \epsilon_{xx}\\ \epsilon_{yy}\\ \epsilon_{xy} \end{array}\right\},\;\epsilon_{n}=\left\{\begin{array}{c} \gamma_{xz}\\ \gamma_{yz}\\ \gamma_{zz} \end{array}\right\}.$$

The hypothesis of small displacements helps to use a linear strain–displacement relation:

(5) $$\begin{array}{c} \epsilon_{p}=D_{p}u\;,\\ \epsilon_{n}=\left(D_{n\Omega }+D_{nz}\right)u\;, \end{array}$$

where $D_{p},D_{n\Omega }$ and $D_{nz}$ are the following differential operators:

(6) $$D_{p}=\left[\begin{array}{ccc} \;\frac{\partial }{\partial x} & 0 & 0\\ \;0 & \frac{\partial }{\partial y} & 0\\ \frac{\partial }{\partial y} & \frac{\partial }{\partial x} & 0 \end{array}\right],\;D_{n\Omega }=\left[\begin{array}{ccc} \;0 & 0 & \frac{\partial }{\partial x}\\ \;0 & 0 & \frac{\partial }{\partial y}\\ 0 & 0 & 0 \end{array}\right],\;D_{nz}=\left[\begin{array}{ccc} \;\frac{\partial }{\partial z} & 0 & 0\\ \;0 & \frac{\partial }{\partial z} & 0\\ 0 & 0 & \frac{\partial }{\partial z} \end{array}\right].$$

The stress vector is expressed in a similar manner:

(7) $$\sigma_{p}=\left\{\begin{array}{c} \sigma_{xx}\\ \sigma_{yy}\\ \sigma_{xy} \end{array}\right\},\;\sigma_{n}=\left\{\begin{array}{c} \sigma_{xz}\\ \sigma_{yz}\\ \sigma_{zz} \end{array}\right\}.$$

Hooke’s law reads:

(8) $$\begin{array}{c} \sigma_{p}={\tilde{C}}_{pp}\epsilon_{p}+{\tilde{C}}_{pn}\epsilon_{n}\;,\\ \sigma_{n}={\tilde{C}}_{np}\epsilon_{p}+{\tilde{C}}_{nn}\epsilon_{n}\;, \end{array}$$

where the terms ${\tilde{C}}_{pp}$, ${\tilde{C}}_{pn}$, ${\tilde{C}}_{np}$ and ${\tilde{C}}_{nn}$ are subcomponents of a material stiffness matrix $\tilde{C}$ according to the stress and strain ordering in Equations (4) and (7), where the fibers lay in Ω and where they are not, in general, aligned with the *x*-axis. C stands for the stiffness matrix in the global reference system, and its components can be written in terms of the Young’s moduli $E_{L}$ and $E_{T}$, shear moduli $G_{LT}$ and $G_{TT}$ and Poisson’s ratios $\nu_{LT}$ and $\nu_{TT}$, where subscripts *L* and *T* stand for the directions parallel and perpendicular to the fibers, respectively. For further details, see Reddy [38].

### 2.1. Variable Stiffness Composite Plates

Laminated VAT structures are considered in this work. For this reason, the material stiffness coefficients can change layer-wise along the thickness and pointwise along the in-plane directions. The mapping of C into $\tilde{C}$ reads:

(9) $$\tilde{C}=TCT^{T}\;.$$

Superscript *T* stands for the transpose operator. The matrix T represents a rotation matrix that depends on an in-plane rotation angle θ. For the sake of brevity, the components of $\tilde{C}$ and T are not reported here; they can be found in Reddy [38]. In a laminated VAT, the rotation angle θ is a bi-dimensional field in Ω. In this work, two different variation laws are considered for θ, a linear variation law and a parabolic one. The linear law can be expressed according to the following formula:

(10) $$\theta \left(\alpha \right)=\Phi +T_{0}+\frac{T_{1}-T_{0}}{d}\left|\alpha \right|\;.$$

The angle Φ describes the original direction along which θ varies, and α is a generic spatial variable defined as:

(11) $$\alpha =x^{\prime }\cos\left(\Phi \right)+y^{\prime }\sin\left(\Phi \right)\;.$$

**Figure 2:**
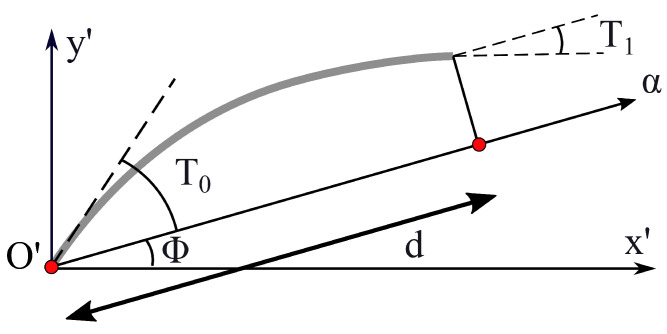
Example of in-plane fiber orientation.

$x^{\prime }$ and $y^{\prime }$ denote a generic in-plane reference system used for describing a fiber path, where θ is measured. The introduction of a new reference system is useful in order to represent the local fiber orientation independently from the global reference system identified by axes *x* and *y*. $T_{0}$ and $T_{1}$ are the angles between the α-axis and the tangent to a fiber for α equal to zero and *d*, respectively; see Figure 2.

As shown in the Figure 2, the fiber angle is always measured with respect to the $x^{\prime }$-axis, and it can change along a generic direction α, defined as a combination of $x^{\prime }$ and $y^{\prime }$ depending on the angle Φ. Further details about the fiber linear variation law can be found in Gürdal et al. [39]. The parabolic law can be expressed according to the following equation:

(12) $$\theta \left(\alpha \right)=\Phi +T_{0}+\tan^{-1}\left(\gamma \frac{\alpha }{d}\right)\;.$$

The parameter γ is used to control the shape of the parabola, and it is related to the final fiber angle $T_{1}$ as $T_{1}=\tan^{-1}\left(\pm \gamma \right)$. More details about the parabolic fiber path can be found in Hachemi et al. [9] and Honda et al. [40]. The following notation, based upon the above introduced parameters, is used in order to describe the in-plane linear and parabolic fiber behavior: $\Phi <T_{0},T_{1}>$.

### 2.2. Variational Statements

PVD and RMVT variational statements are considered to derive the governing equations for the free vibration problem for a laminated VAT plate. The fundamental distinction is that the RMVT considers the vector of the out-of-plane stresses $\sigma_{n}$ as a primary unknown, whereas the PVD considers only displacements as primary variables. For the PVD case, the following variational statement applies:

(13) $$\int_{\Omega }\int_{H}\left(\delta \epsilon_{pG}^{T}\;\sigma_{pH}+\delta \epsilon_{nG}^{T}\;\sigma_{nH}\right)\;dz\;d\Omega +\delta L_{in}=0\;,$$

where the subscript *G* refers to the components obtained from the geometrical relations in Equation (5), and subscript *H* refers to the components obtained from Hooke’s law in Equation (8). $L_{in}$ is the virtual work of the inertial forces, and δ stands for a virtual variation. For the RMVT case, the variational statement is:

(14) $$\int_{\Omega }\int_{H}\left[\delta \epsilon_{pG}^{T}\;\sigma_{pH}+\delta \epsilon_{nG}^{T}\;\sigma_{nM}+\delta \sigma_{nM}^{T}\left(\epsilon_{nG}-\epsilon_{nH}\right)\right]\;dz\;d\Omega +\delta L_{in}=0\;.$$

The *M* subscript refers to the transverse stress components considered as primary unknowns in the mixed formulation. For the RMVT formulation, Hooke’s law is rewritten as follows:

(15) $$\begin{array}{c} \sigma_{pH}={\hat{C}}_{pp}\epsilon_{pG}+{\hat{C}}_{pn}\sigma_{nM}\;,\\ \epsilon_{nH}={\hat{C}}_{np}\epsilon_{pG}+{\hat{C}}_{nn}\sigma_{nM}\;, \end{array}$$

where ${\hat{C}}_{pp}$, ${\hat{C}}_{pn}$, ${\hat{C}}_{np}$ and ${\hat{C}}_{nn}$ are (see Carrera and Demasi [35]):

(16) $$\begin{array}{c} {\hat{C}}_{pp}={\tilde{C}}_{pp}-{\tilde{C}}_{pn}{\tilde{C}}_{nn}^{-1}{\tilde{C}}_{np}\;,\\ {\hat{C}}_{pn}={\tilde{C}}_{pn}{\tilde{C}}_{nn}^{-1}\;,\\ {\hat{C}}_{np}=-{\tilde{C}}_{nn}^{-1}{\tilde{C}}_{np}\;,\\ {\hat{C}}_{nn}={\tilde{C}}_{nn}^{-1}\;. \end{array}$$

The superscript “−1” indicates the inverse of a matrix. The inertial work can be expressed as:

(17) $$\delta L_{in}=\int_{\Omega }\int_{H}\delta u^{T}\rho \;\ddot{u}\;d\Omega \;dz\;,$$

where ρ is the plate material density, and $\ddot{u}$ represents the acceleration vector.

### 2.3. Kinematic Assumptions

CUF uses an axiomatic approach along the through-the-thickness direction to represent the primary unknowns; see Carrera [22]. The generic unknown component $f=f\left(x,y,z\right)$ is approximated as:

(18) $$f\left(x,y,z\right)=F_{\tau }\left(z\right)g_{\tau }\left(x,y\right)\;,\;\;\;\tau =0,\;1,\;\cdots ,\;N\;,$$

where *f* is a displacement component in a formulation derived from the PVD, but it can also be an out-of-plane stress component when a RMVT formulation is considered. $F_{\tau }$ is an approximation function in H, and $g_{\tau }$ is an unknown two-dimensional function in Ω. According to Einstein’s notation, a twice-repeated index implies a sum over the index range. Finally, *N* is the approximation order. Both *N* and $F_{\tau }$ are a priori defined. This feature of CUF helps to obtain multiple theories in the same formulation. Within CUF, ESL or LW models can also be obtained depending on the support of $F_{\tau }$. In an ESL model $F_{\tau }:H\mapsto R$, whereas for a LW model $F_{\tau }:H^{k}\mapsto R$ where $H^{k}=\left\{z^{k}\;:\;\frac{2z^{k}}{h^{k}}\in \left[-1,1\right]\right\}$ such that $H=\overset{N_{l}}{\underset{k=1}{⋃}}H^{k}$ and $H^{k}\cap H^{k^{\prime }}=\emptyset$ for $k\neq k^{\prime }$ with *k*, $k^{\prime }=1,\;2,\;\cdots ,\;N_{l}$, where $N_{l}$ is the total number of laminae, and $h^{k}$ is the thickness of a generic *k* lamina such that $k=\sum_{k=1}^{N_{l}}h^{k}$. The number of unknowns in the ESL case is independent of the number of layers in the lamination since the approximation is imposed globally over H. The total stiffness contributions can be seen as a weighted average of each layer stiffness along the thickness. Maclaurin’s series approximation is considered for the ESL models as a linear combination of the power functions:

(19) $$F_{\tau }\left(z\right)=z^{\tau }\;,\;\;\;\tau =0,\;1,\;\cdots ,\;N\;,$$

where *N* is the expansion order. The computational cost of ESL models depends on *N* only, and for a given *N*, it is lower than a LW model since this latter model depends on the total number of layers in the lamination. ESL models are suitable for relatively thick laminates. However, they are unable to accurately predict the behavior of thick plates with a high degree of anisotropy. ESL models have $C^{\infty }$-continuity over H because of the used approximation functions, whereas laminated composites present a $C^{0}$-continuity since the interface between the two consecutive layers of the different materials introduces a change in the slope of the displacements (also known as zig-zag displacement through-the-thickness variation). This behavior can be accommodated within an ESL theory by means of Murakami’s function. This approach is not considered here; for more details, refer to Carrera [41]. In an LW model, the kinematics of each layer are formulated independently:

(20) $$f^{k}\left(x,y,z\right)=F_{b}\left(z\right)g_{b}^{k}\left(x,y\right)+F_{r}\left(z\right)g_{r}^{k}\left(x,y\right)+F_{t}\left(z\right)g_{t}^{k}\left(x,y\right)\;,\;\;\;r=2,\;\cdots ,\;N\;,$$

where subscripts *b* and *t* stand for the bottom and top layers, respectively. Congruence at the interface is retrieved via a through-the-thickness assembly procedure similar to that used in the finite element method. For this reason, Lagrange polynomials (which ensure partition of unity), or the following linear combination of Legendre polynomials, which are represented as:

(21) $$F_{t}\left(z(\xi^{k})\right)=\frac{P_{0}+P_{1}}{2},\;\;\;F_{b}\left(z(\xi^{k})\right)=\frac{P_{0}-P_{1}}{2},\;\;\;F_{r}\left(z(\xi^{k})\right)=P_{r}-P_{r-2},\;\;\;r=2,\;\cdots ,\;N$$

are typically used as approximation functions over $H^{k}$. The use of Lagrange or Legendre polynomials along the thickness changes according to the used model, and this is specified at the end of the next subsection. In Equation (21), $\xi^{k}=\frac{2z^{k}}{h^{k}}\in \left[-1,1\right]$ and $P_{i}=P_{i}\left(\xi^{k}\right)$ are an *i*-order Legendre polynomial. Equation (21) creates a base where $F_{t}$ and $F_{b}$ are the two linear Lagrange polynomials, and $F_{r}$ is a kind of *p*-version-enriching function since it does not contribute to a base linear combination for $\xi^{k}=\pm 1$, being, by definition, $F_{r}\left(\pm 1\right)=0$. Since LW base functions have local support, inter-layer $C^{0}$-continuity for layers made of different materials is ensured, but the computational costs are higher than for ESL models.

### 2.4. Acronym System

**Figure 3:**
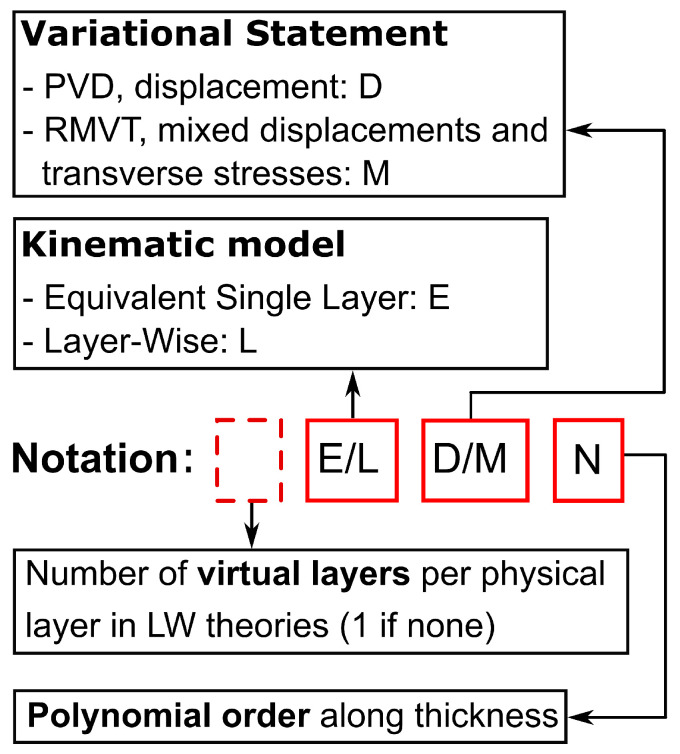
Acronym system.

An acronym system is used in order to identify all the derived theories. Figure 3 shows this system.

The first letter addresses the approximation level that is applied: ‘E’ denotes the ESL models, whereas ‘L’ denotes the LW models. The second letter denotes the variational statement: PVD or RMVT are denoted by ‘D’ or ‘M’, respectively. The last number is the order of expansion along the plate thickness. A number at the beginning of the acronym, when present, indicates how many virtual layers have been used to approximate each physical layer in an LW model to improve the results for a given approximation order. If this number is not present, only one virtual layer has been used to represent each physical layer.

As an example, in EDN models, the displacement field can be expressed as:

(22) $$\begin{array}{c} \;u_{x}=u_{x0}+u_{x1}z+u_{x2}z^{2}+\;\cdots +u_{xN}z^{N}\;,\\ \;u_{y}=u_{y0}+u_{y1}z+u_{y2}z^{2}+\;\cdots +u_{yN}z^{N}\;,\\ u_{z}=u_{z0}+u_{z1}z+u_{z2}z^{2}+\;\cdots +u_{zN}z^{N}\;. \end{array}$$

In vector form:

(23) $$u=F_{0}u_{0}+F_{1}u_{1}+\;\cdots +F_{N}u_{N}=F_{\tau }u_{\tau }\;,\;\;\;\tau =0,\;1,\;\cdots ,\;N\;,$$

where $F_{\tau }=z^{\tau }$ and $u_{\tau }=u_{\tau }\left(x,y\right)$. Additionally, classical theories can be taken into account. Classical lamination theory (CLT) and first-order shear deformation theory are obtained as a particular case of a first-order ESL theory. FSDT is obtained through the penalization of the $uz_{1}$ term, while for CLT, transverse shear stresses are disregarded by using a fictitiously high value of the material shear moduli. The material stiffness matrix needs to be reduced in a plane-stress sense to overcome thickness locking.

For LDN solutions, only displacements are considered as the primary variables:

(24) $$u^{k}=F_{0}u_{0}^{k}+F_{1}u_{1}^{k}+\cdots +F_{N}u_{N}^{k}=F_{\tau }u_{\tau }^{k}\;,\;\;\;\;\;\;\;\;\tau =0,\;1,\;\cdots ,\;N\;,\;k=1,\;2,\;\cdots ,\;N_{l}\;.$$

For LMN solutions, transverse stresses are treated as primary variables. The transverse stress field can be expressed as:

(25) $$\sigma_{n}^{k}=F_{0}\sigma_{0}^{k}+F_{1}\sigma_{1}^{k}+\cdots +F_{N}\sigma_{N}^{k}=F_{\tau }\sigma_{\tau }^{k}\;,\;\;\;\;\;\;\;\;\tau =0,\;1,\;\cdots ,\;N\;,\;k=1,\;2,\;\cdots ,\;N_{l}\;.$$

It can be observed that ESL theories can be considered as a particular case for LW theories. While in the first case the integration along the thickness is performed in order to represent composite properties through a unitary layer, for the second case, the integration is computed layer by layer. This helps to represent the kinematics of each layer separately for LW models. LDN solutions are obtained with Lagrange polynomials with equally spaced nodes, whereas LMN ones are obtained with Legendre polynomials.

### 2.5. FE Stiffness Matrices

As far as a FEM solution is concerned, the in-plane domain is discretized into $N_{e}$ subdomains such as $\Omega =\overset{N_{e}}{\underset{e=1}{⋃}}\Omega_{e}$ and $\Omega_{e}\cap \Omega^{e^{\prime }}=\emptyset$ for $e\neq e^{\prime }$. Shape functions are then introduced for the approximation of the variation over $\Omega_{e}$. In the case of a bi-dimensional model, Equation (18) becomes:

(26) $$f\left(x,y,z\right)=F_{\tau }\left(z\right)N_{i}\left(x,y\right)g_{\tau i}\;,\;\;\;\tau =0,\;1,\;\cdots ,\;N\;,\;\;\;i=1,\;\cdots ,\;N_{n}\;,$$

where $N_{i}$ stands for the shape functions, and $N_{n}$ is the number of nodes in the used finite element. Classical Lagrange shape functions are used. They are not presented here for the sake of brevity, but they can be found in Bathe [42]. FE stiffness matrices are obtained by the weak form of the variational principles. In the PVD case, considering Equation (26), the displacement field can be written as:

(27) $$u=F_{\tau }N_{i}\left\{\begin{array}{c} q_{x\tau i}\\ q_{y\tau i}\\ q_{z\tau i} \end{array}\right\}=F_{\tau }N_{i}q_{\tau i}\;.$$

Through the substitution of Equations (5), (8) and (27) into Equation (13), the weak PVD form can be obtained:

(28) $$\begin{array}{cc} \int_{\Omega_{e}} & \delta q_{\tau i}^{T}[D_{p}^{T}\left(N_{i}I\right){\tilde{Z}}_{pp}^{\tau s}D_{p}\left(N_{j}I\right)+D_{p}^{T}\left(N_{i}I\right){\tilde{Z}}_{pn}^{\tau s}D_{n\Omega }\left(N_{j}I\right)+D_{p}^{T}\left(N_{i}I\right){\tilde{Z}}_{pn}^{\tau s{,}_{z}}\left(N_{j}I\right)\\ + & D_{n\Omega }^{T}\left(N_{i}I\right){\tilde{Z}}_{np}^{\tau s}D_{p}\left(N_{j}I\right)+D_{n\Omega }^{T}\left(N_{i}I\right){\tilde{Z}}_{nn}^{\tau s}D_{n\Omega }\left(N_{j}I\right)+D_{n\Omega }^{T}\left(N_{i}I\right){\tilde{Z}}_{nn}^{\tau s{,}_{z}}\left(N_{j}I\right)\\ + & \left(N_{i}I\right){\tilde{Z}}_{np}^{\tau {,}_{z}s}D_{p}\left(N_{j}I\right)+\left(N_{i}I\right){\tilde{Z}}_{nn}^{\tau {,}_{z}s}D_{n\Omega }\left(N_{j}I\right)+\left(N_{i}I\right){\tilde{Z}}_{nn}^{\tau {,}_{z}s{,}_{z}}\left(N_{j}I\right)]\\ & q_{sj}d\Omega =-\int_{\Omega_{e}}\delta q_{\tau i}^{T}\left(N_{i}I\right)\rho E_{\tau s}\left(N_{j}I\right){\ddot{q}}_{sj}d\Omega \;, \end{array}$$

where:

(29) $$({\tilde{Z}}_{wr}^{\tau s},{\tilde{Z}}_{wr}^{\tau {,}_{z}s},{\tilde{Z}}_{wr}^{\tau s{,}_{z}},{\tilde{Z}}_{wr}^{\tau {,}_{z}s{,}_{z}})=({\tilde{C}}_{wr}E_{\tau s},{\tilde{C}}_{wr}E_{\tau {,}_{z}s},{\tilde{C}}_{wr}E_{\tau s{,}_{z}},{\tilde{C}}_{wr}E_{\tau {,}_{z}s{,}_{z}}):\;w,r=p,n\;,$$

(30) $$(E_{\tau s},E_{\tau {,}_{z}s},E_{\tau s{,}_{z}},E_{\tau {,}_{z}s{,}_{z}})=\int_{H}(F_{\tau }F_{s},F_{\tau {,}_{z}}F_{s},F_{\tau }F_{s{,}_{z}},F_{\tau {,}_{z}}F_{s{,}_{z}})dz\;.$$

An axis coordinate as comma-preceded subscript stands for a derivative in that coordinate direction. In compact vector form, Equation (28) reads:

(31) $$\delta q_{\tau i}^{T}K^{\tau sij}q_{sj}=-\delta q_{\tau i}^{T}M^{\tau sij}{\ddot{q}}_{sj}\;,$$

where $K^{\tau sij}$ and $M^{\tau sij}\in R^{3\times 3}$ are fundamental nuclei (FN) of the stiffness and mass matrices, respectively. Through the cycles on the indices τ, *s*, *i* and *j*, it is possible to build the stiffness and mass matrices of a finite element. The components of the stiffness FN for the PVD case can be written as:

(32) $$\begin{array}{cc} \; & K_{xx}^{\tau sij}=\int_{\Omega_{e}}\left({\tilde{Z}}_{pp11}^{\tau s}N_{j{,}_{x}}N_{i{,}_{x}}+{\tilde{Z}}_{pp16}^{\tau s}N_{j{,}_{y}}N_{i{,}_{x}}+{\tilde{Z}}_{pp16}^{\tau s}N_{j{,}_{x}}N_{i{,}_{y}}+{\tilde{Z}}_{pp66}^{\tau s}N_{j{,}_{y}}N_{i{,}_{y}}+{\tilde{Z}}_{nn44}^{\tau {,}_{z}s{,}_{z}}N_{j}N_{i}\right)d\Omega \;,\\ \; & K_{xy}^{\tau sij}=\int_{\Omega_{e}}\left({\tilde{Z}}_{pp12}^{\tau s}N_{j{,}_{y}}N_{i{,}_{x}}+{\tilde{Z}}_{pp16}^{\tau s}N_{j{,}_{x}}N_{i{,}_{x}}+{\tilde{Z}}_{pp26}^{\tau s}N_{j{,}_{y}}N_{i{,}_{y}}+{\tilde{Z}}_{pp66}^{\tau s}N_{j{,}_{x}}N_{i{,}_{y}}+{\tilde{Z}}_{nn45}^{\tau {,}_{z}s{,}_{z}}N_{j}N_{i}\right)d\Omega \;,\\ \; & K_{xz}^{\tau sij}=\int_{\Omega_{e}}\left({\tilde{Z}}_{pn13}^{\tau s{,}_{z}}N_{j}N_{i{,}_{x}}+{\tilde{Z}}_{pn36}^{\tau s{,}_{z}}N_{j}N_{i{,}_{y}}+{\tilde{Z}}_{nn44}^{\tau {,}_{z}s}N_{j{,}_{x}}N_{i}+{\tilde{Z}}_{nn45}^{\tau {,}_{z}s}N_{j{,}_{y}}N_{i}\right)d\Omega \;,\\ \; & K_{yx}^{\tau sij}=\int_{\Omega_{e}}\left({\tilde{Z}}_{pp12}^{\tau s}N_{j{,}_{x}}N_{i{,}_{y}}+{\tilde{Z}}_{pp26}^{\tau s}N_{j{,}_{y}}N_{i{,}_{y}}+{\tilde{Z}}_{pp16}^{\tau s}N_{j{,}_{x}}N_{i{,}_{x}}+{\tilde{Z}}_{pp66}^{\tau s}N_{j{,}_{y}}N_{i{,}_{x}}+{\tilde{Z}}_{nn45}^{\tau {,}_{z}s{,}_{z}}N_{j}N_{i}\right)d\Omega \;,\\ \; & K_{yy}^{\tau sij}=\int_{\Omega_{e}}\left({\tilde{Z}}_{pp22}^{\tau s}N_{j{,}_{y}}N_{i{,}_{y}}+{\tilde{Z}}_{pp26}^{\tau s}N_{j{,}_{x}}N_{i{,}_{y}}+{\tilde{Z}}_{pp26}^{\tau s}N_{j{,}_{y}}N_{i{,}_{x}}+{\tilde{Z}}_{pp66}^{\tau s}N_{j{,}_{x}}N_{i{,}_{x}}+{\tilde{Z}}_{nn55}^{\tau {,}_{z}s{,}_{z}}N_{j}N_{i}\right)d\Omega \;,\\ \; & K_{yz}^{\tau sij}=\int_{\Omega_{e}}\left({\tilde{Z}}_{pn23}^{\tau s{,}_{z}}N_{j}N_{i{,}_{y}}+{\tilde{Z}}_{pn36}^{\tau s{,}_{z}}N_{j}N_{i{,}_{x}}+{\tilde{Z}}_{nn45}^{\tau {,}_{z}s}N_{j{,}_{x}}N_{i}+{\tilde{Z}}_{nn55}^{\tau {,}_{z}s}N_{j{,}_{y}}N_{i}\right)d\Omega \;,\\ \; & K_{zx}^{\tau sij}=\int_{\Omega_{e}}\left({\tilde{Z}}_{nn44}^{\tau s{,}_{z}}N_{j}N_{i{,}_{x}}+{\tilde{Z}}_{nn45}^{\tau s{,}_{z}}N_{j}N_{i{,}_{y}}+{\tilde{Z}}_{np13}^{\tau {,}_{z}s}N_{j{,}_{x}}N_{i}+{\tilde{Z}}_{np36}^{\tau {,}_{z}s}N_{j{,}_{y}}N_{i}\right)d\Omega \;,\\ \; & K_{zy}^{\tau sij}=\int_{\Omega_{e}}\left({\tilde{Z}}_{nn45}^{\tau s{,}_{z}}N_{j}N_{i{,}_{x}}+{\tilde{Z}}_{nn55}^{\tau s{,}_{z}}N_{j}N_{i{,}_{y}}+{\tilde{Z}}_{np23}^{\tau {,}_{z}s}N_{j{,}_{y}}N_{i}+{\tilde{Z}}_{np36}^{\tau {,}_{z}s}N_{j{,}_{x}}N_{i}\right)d\Omega \;,\\ \; & K_{zz}^{\tau sij}=\int_{\Omega_{e}}\left({\tilde{Z}}_{nn44}^{\tau s}N_{j{,}_{x}}N_{i{,}_{x}}+{\tilde{Z}}_{nn45}^{\tau s}N_{j{,}_{y}}N_{i{,}_{x}}+{\tilde{Z}}_{nn45}^{\tau s}N_{j{,}_{x}}N_{i{,}_{y}}+{\tilde{Z}}_{nn55}^{\tau s}N_{j{,}_{y}}N_{i{,}_{y}}+{\tilde{Z}}_{nn33}^{\tau {,}_{z}s{,}_{z}}N_{j}N_{i}\right)d\Omega \;. \end{array}$$

The mass FN can be written as:

(33) $$M^{\tau sij}=\int_{\Omega_{e}}\left(N_{i}I\right)\rho E_{\tau s}\left(N_{j}I\right)d\Omega \;.$$

It is possible to observe that $M^{\tau sij}$ is a diagonal matrix and that since the plate density is assumed to be constant, the term $\rho E_{\tau s}$ can be placed outside the integral.

In the RMVT case, transverse stresses are a priori approximated:

(34) $$\sigma_{n}=F_{\tau }N_{i}\left\{\begin{array}{c} g_{xz\tau i}\\ g_{yz\tau i}\\ g_{zz\tau i} \end{array}\right\}=F_{\tau }N_{i}g_{\tau i}\;.$$

Through the substitution of Equations (5), (15), (27) and (34) into Equation (14), the RMVT weak form can be obtained:

(35) $$\begin{array}{cc} \int_{\Omega_{e}} & \delta q_{\tau i}^{T}[D_{p}^{T}\left(N_{i}I\right){\hat{Z}}_{pp}^{\tau s}D_{p}\left(N_{j}I\right)]q_{sj}+\delta q_{\tau i}^{T}[D_{p}^{T}\left(N_{i}I\right){\hat{Z}}_{pn}^{\tau s}\left(N_{j}I\right)+D_{n\Omega }^{T}\left(N_{i}I\right)\left(E_{\tau s}I\right)\left(N_{j}I\right)\\ + & \left(N_{i}I\right)\left(E_{\tau {,}_{z}s}I\right)\left(N_{j}I\right)]g_{sj}+\delta g_{\tau i}^{T}[\left(N_{i}I\right)\left(E_{\tau s}I\right)D_{n\Omega }\left(N_{j}I\right)+\left(N_{i}I\right)\left(E_{\tau s{,}_{z}}I\right)\left(N_{j}I\right)\\ - & \left(N_{i}I\right){\hat{Z}}_{np}^{\tau s}D_{p}\left(N_{j}I\right)]q_{sj}-\delta g_{\tau i}^{T}\left(N_{i}I\right){\hat{Z}}_{nn}^{\tau s}\left(N_{j}I\right)g_{sj}d\Omega =-\int_{\Omega_{e}}\delta q_{\tau i}^{T}\left(N_{i}I\right)\rho E_{\tau s}\left(N_{j}I\right){\ddot{q}}_{sj}d\Omega \;, \end{array}$$

where:

(36) $$({\hat{Z}}_{wr}^{\tau s},{\hat{Z}}_{wr}^{\tau {,}_{z}s},{\hat{Z}}_{wr}^{\tau s{,}_{z}},{\hat{Z}}_{wr}^{\tau {,}_{z}s{,}_{z}})=({\hat{C}}_{wr}E_{\tau s},{\hat{C}}_{wr}E_{\tau {,}_{z}s},{\hat{C}}_{wr}E_{\tau s{,}_{z}},{\hat{C}}_{wr}E_{\tau {,}_{z}s{,}_{z}}):\;w,r=p,n\;.$$

In a compact form:

(37) $$\begin{array}{cc} \; & \delta q_{\tau i}^{T}K_{uu}^{\tau sij}q_{sj}+\delta q_{\tau i}^{T}K_{u\sigma }^{\tau sij}g_{sj}=-\delta q_{\tau i}^{T}M^{\tau sij}{\ddot{q}}_{sj}\;,\\ \; & \delta g_{\tau i}^{T}K_{\sigma u}^{\tau sij}q_{sj}+\delta g_{\tau i}^{T}K_{\sigma \sigma }^{\tau sij}g_{sj}=0\;. \end{array}$$

In this case, four fundamental nuclei are obtained. The components of the FN for the RMVT case can be written as:

(38) $$\begin{array}{cc} \; & K_{uuxx}^{\tau sij}=\int_{\Omega_{e}}\left({\hat{Z}}_{pp11}^{\tau s}N_{j{,}_{x}}N_{i{,}_{x}}+{\hat{Z}}_{pp31}^{\tau s}N_{j{,}_{x}}N_{i{,}_{y}}+{\hat{Z}}_{pp13}^{\tau s}N_{j{,}_{y}}N_{i{,}_{x}}+{\hat{Z}}_{pp33}^{\tau s}N_{j{,}_{y}}N_{i{,}_{y}}\right)d\Omega \;,\\ \; & K_{uuxy}^{\tau sij}=\int_{\Omega_{e}}\left({\hat{Z}}_{pp12}^{\tau s}N_{j{,}_{y}}N_{i{,}_{x}}+{\hat{Z}}_{pp32}^{\tau s}N_{j{,}_{y}}N_{i{,}_{y}}+{\hat{Z}}_{pp13}^{\tau s}N_{j{,}_{x}}N_{i{,}_{x}}+{\hat{Z}}_{pp33}^{\tau s}N_{j{,}_{x}}N_{i{,}_{y}}\right)d\Omega \;,\\ \; & K_{uuyx}^{\tau sij}=\int_{\Omega_{e}}\left({\hat{Z}}_{pp21}^{\tau s}N_{j{,}_{x}}N_{i{,}_{y}}+{\hat{Z}}_{pp31}^{\tau s}N_{j{,}_{x}}N_{i{,}_{x}}+{\hat{Z}}_{pp23}^{\tau s}N_{j{,}_{y}}N_{i{,}_{y}}+{\hat{Z}}_{pp33}^{\tau s}N_{j{,}_{y}}N_{i{,}_{x}}\right)d\Omega \;,\\ \; & K_{uuyy}^{\tau sij}=\int_{\Omega_{e}}\left({\hat{Z}}_{pp22}^{\tau s}N_{j{,}_{y}}N_{i{,}_{y}}+{\hat{Z}}_{pp32}^{\tau s}N_{j{,}_{y}}N_{i{,}_{x}}+{\hat{Z}}_{pp23}^{\tau s}N_{j{,}_{x}}N_{i{,}_{y}}+{\hat{Z}}_{pp33}^{\tau s}N_{j{,}_{x}}N_{i{,}_{x}}\right)d\Omega \;,\\ \; & K_{uuxz}^{\tau sij}=0\;,\;\;\;\;K_{uuyz}^{\tau sij}=0\;,\;\;\;\;K_{uuzx}^{\tau sij}=0\;,\;\;\;\;K_{uuzy}^{\tau sij}=0\;,\;\;\;\;K_{uuzz}^{\tau sij}=0\;,\\ \; & K_{u\sigma xx}^{\tau sij}=\int_{\Omega_{e}}\left(E_{\tau {,}_{z}s}N_{j}N_{i}\right)d\Omega \;,\;\;\;\;K_{u\sigma xz}^{\tau sij}=\int_{\Omega_{e}}\left({\hat{Z}}_{pn13}^{\tau s}N_{j}N_{i{,}_{x}}+{\hat{Z}}_{pn33}^{\tau s}N_{j}N_{i{,}_{y}}\right)d\Omega \;,\\ \; & K_{u\sigma yy}^{\tau sij}=\int_{\Omega_{e}}\left(E_{\tau {,}_{z}s}N_{j}N_{i}\right)d\Omega \;,\;\;\;\;K_{u\sigma yz}^{\tau sij}=\int_{\Omega_{e}}\left({\hat{Z}}_{pn23}^{\tau s}N_{j}N_{i{,}_{y}}+{\hat{Z}}_{pn33}^{\tau s}N_{j}N_{i{,}_{x}}\right)d\Omega \;,\\ \; & K_{u\sigma zx}^{\tau sij}=\int_{\Omega_{e}}\left(E_{\tau s}N_{j}N_{i{,}_{x}}\right)d\Omega \;,\;\;\;\;K_{u\sigma zy}^{\tau sij}=\int_{\Omega_{e}}\left(E_{\tau s}N_{j}N_{i{,}_{y}}\right)d\Omega \;,\;\;\;\;K_{u\sigma zz}^{\tau sij}=\int_{\Omega_{e}}\left(E_{\tau {,}_{z}s}N_{j}N_{i}\right)d\Omega \;,\\ \; & K_{u\sigma xy}^{\tau sij}=0\;,\;\;\;\;K_{u\sigma yx}^{\tau sij}=0\;,\\ \; & K_{\sigma uxx}^{\tau sij}=\int_{\Omega_{e}}\left(E_{\tau s{,}_{z}}N_{j}N_{i}\right)d\Omega \;,\;\;\;\;K_{\sigma uxz}^{\tau sij}=\int_{\Omega_{e}}\left(E_{\tau s}N_{j{,}_{x}}N_{i}\right)d\Omega \;,\;\;\;\;K_{\sigma uyy}^{\tau sij}=\int_{\Omega_{e}}\left(E_{\tau s{,}_{z}}N_{j}N_{i}\right)d\Omega \;,\\ \; & K_{\sigma uyz}^{\tau sij}=\int_{\Omega_{e}}\left(E_{\tau s}N_{j{,}_{y}}N_{i}\right)d\Omega \;,\;\;\;\;K_{\sigma uzx}^{\tau sij}=-\int_{\Omega_{e}}\left({\hat{Z}}_{np31}^{\tau s}N_{j{,}_{x}}N_{i}-{\hat{Z}}_{np33}^{\tau s}N_{j{,}_{y}}N_{i}\right)d\Omega \;,\\ \; & K_{\sigma uzy}^{\tau sij}=-\int_{\Omega_{e}}\left({\hat{Z}}_{np32}^{\tau s}N_{j{,}_{y}}N_{i}-{\hat{Z}}_{np33}^{\tau s}N_{j{,}_{x}}N_{i}\right)d\Omega \;,\;\;\;\;K_{\sigma uzz}^{\tau sij}=\int_{\Omega_{e}}\left(E_{\tau s{,}_{z}}N_{j}N_{i}\right)d\Omega \;,\\ \; & K_{\sigma uxy}^{\tau sij}=0\;,\;\;\;\;K_{\sigma uyx}^{\tau sij}=0\;,\\ \; & K_{\sigma \sigma xx}^{\tau sij}=-\int_{\Omega_{e}}\left({\hat{Z}}_{nn11}^{\tau s}N_{j}N_{i}\right)d\Omega \;,\;\;\;\;K_{\sigma \sigma xy}^{\tau sij}=-\int_{\Omega_{e}}\left({\hat{Z}}_{nn12}^{\tau s}N_{j}N_{i}\right)d\Omega \;,\\ \; & K_{\sigma \sigma yx}^{\tau sij}=-\int_{\Omega_{e}}\left({\hat{Z}}_{nn21}^{\tau s}N_{j}N_{i}\right)d\Omega \;,\;\;\;\;K_{\sigma \sigma xx}^{\tau sij}=-\int_{\Omega_{e}}\left({\hat{Z}}_{nn22}^{\tau s}N_{j}N_{i}\right)d\Omega \;,\\ \; & K_{\sigma \sigma xz}^{\tau sij}=0\;,\;\;\;\;K_{\sigma \sigma yz}^{\tau sij}=0\;,\;\;\;\;K_{\sigma \sigma zx}^{\tau sij}=0\;,\;\;\;\;K_{\sigma \sigma zy}^{\tau sij}=0\;,\;\;\;\;K_{\sigma \sigma zz}^{\tau sij}=0\;. \end{array}$$

The mass FN is the same as the PVD case; see Equation (33). Since the in-plane integrals are calculated via Gauss quadrature, it is crucial to consider an appropriate number of Gauss points in accordance with the variational rule of the fiber angle.

## 3. Results and Discussion

Three cases are analyzed in this work: a cantilever monolayer plate, a clamped multilayer plate and a clamped multilayer plate with a central circular cut-out. For each case, a square geometry is considered (a=b=1 m). Parametric studies are performed considering different side-to-thickness ratios (a/h=100,10,5). Material properties are represented in Table 1 for all the considered analyzed cases.

Reference solutions are represented by an Abaqus 3D model where quadratic elements (C3D20R) were used. For CUF solutions, nine-node square elements were used. For each case study, a preliminary convergence analysis was carried out to identify the appropriate mesh for both CUF and Abaqus solutions.

### 3.1. Monolayer Plate

**Figure 4:**
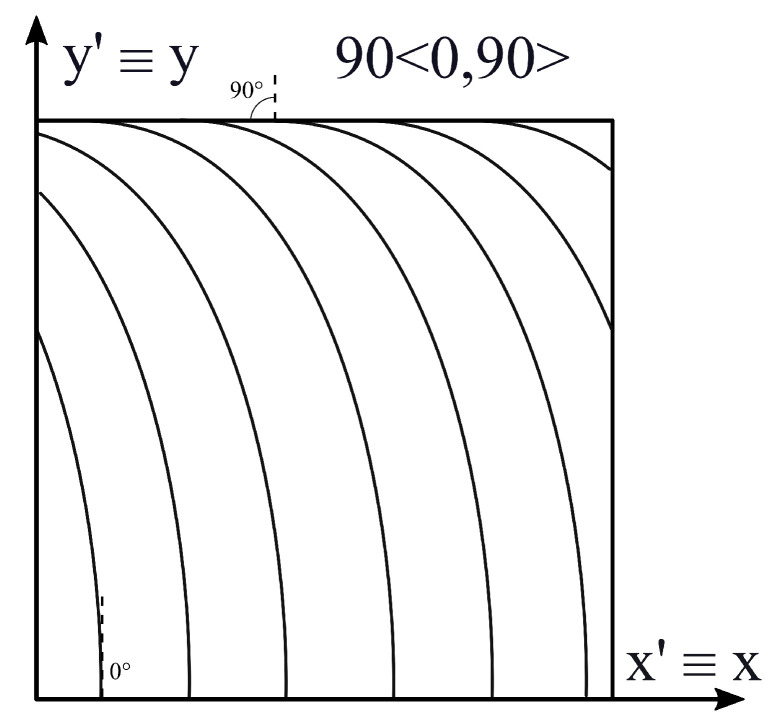
Stacking sequence; case 1.

The first case corresponds to a cantilever monolayer plate with density ρ=1540 kg/m${}^{3}$. For this problem, axes $x^{\prime }$ and $y^{\prime }$ of the angle reference system are coincident with axes *x* and *y* of the plate. This means that the origin of the angle reference system is the same as the global one and that $x^{\prime }$ and $y^{\prime }$ are parallel to *x* and *y*, respectively. It is assumed that the fiber angle is a linear function of $y^{\prime }$; see Equation (10). The length parameter corresponds to d=b, while the direction of fiber variation α corresponds to $y^{\prime }$, which means that $\Phi =90^{\circ }$. In this case, $T_{0}=0^{\circ }$ and $T_{1}=90^{\circ }$. The fiber orientation changes only along $y^{\prime }$ from a value of $\theta \left(0\right)=\Phi +T_{0}=90^{\circ }$ to $\theta \left(b\right)=\Phi +T_{1}=180^{\circ }$. The angle variational law in this case can be expressed as 90<0,90>, and it is presented in Figure 4.

This law has been taken from Viglietti et al. [27]. The reference solution contains 80 elements along each in-plane side and 12 elements along the thickness. The only clamped side of the plate is the one that lies on the xz plane, corresponding to $y^{\prime }=0$. For the CUF results, a 10×10 mesh is considered. Table 2 shows the degrees Of freedom (DOF) for some considered solutions.

It is possible to observe that higher-order CUF models allow for a DOF reduction of one order of magnitude in comparison with the Abaqus 3D reference solution. Table 3 shows the first five natural frequencies for a/h=100.

For this case, classic and higher-order theories show very good approximations of the reference solution, where the maximum difference from the reference solution is 0.4% for the second natural frequency computed via CLT. Table 4 shows the first five natural frequencies for a/h=10.

It is possible to observe that classical and lower-order ESL theories are now less accurate, especially for the prediction of higher frequencies. For example, CLT, FSDT and ED2 models, corresponding to the third natural frequency, present a percentage error equal to 8.1%, 1.0% and 1.1%, respectively. This can be explained by considering that the side-to-thickness ratio a/h=10 corresponds to a thick plate. In this case, higher-order theories are needed to obtain an accurate approximation. Since a moderately thick plate is considered, transverse shear stresses affect the solution. This is the reason that CLT, which neglects those stresses, is less close to the reference solution. The best approximations of plate natural frequencies are given by 2LM2 and 3LM4 mixed theories, which show a maximum percentage error of 0.1% each for the fourth natural frequency. In particular, it is possible to observe that the 2LM2 solution is globally closer to Abaqus in comparison with the 3LD4 solution, even if the last one is characterized by a higher number of degrees of freedom. Table 5 shows the first five natural frequencies for a/h=5.

Because of the low side-to-thickness ratio, a very thick plate is considered, and lower-order theories do not provide a correct prediction of the natural frequencies. For CLT, the sixth mode is the same as the fifth mode of the reference solution, that is, the order of appearance is swapped. In this regard, mode tracking was performed by visually comparing the modes of each proposed solution with those of the reference solution obtained in Abaqus. The corresponding percentage error is as high as 27.1%. On the other hand, a 3LM4 model matches the Abaqus reference results.

### 3.2. Multilayer Plate

**Figure 5:**
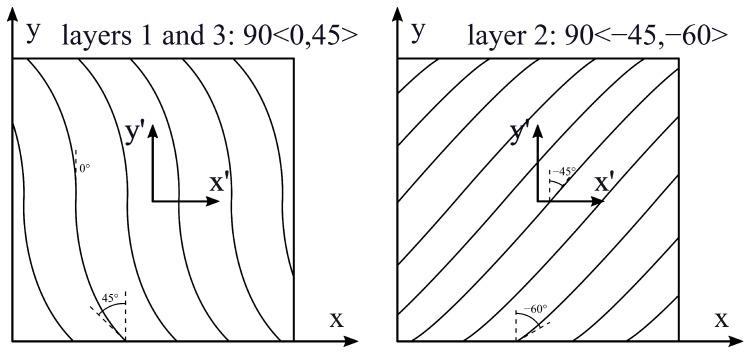
Stacking sequence; case 2.

The second case is taken from Viglietti et al. [27] and corresponds to a multilayer clamped plate with density ρ=1540 kg/m${}^{3}$. The plate is composed of three layers with the same thicknesses. It is assumed that fiber angle is a function of $y^{\prime }$ only, which means that α is parallel to $y^{\prime }$ ($\Phi =90^{\circ }$). In this case, a linear law is considered for the fiber path, according to Equation (10). For this problem, axes $x^{\prime }$ and $y^{\prime }$ of the angle reference system are aligned with axes *x* and *y* of the plate, but their origin is placed on the center of the plate (a/2,b/2). In this case, d=b/2 is considered as the length parameter in Equation (10). $T_{0}$ and $T_{1}$ are set for each layer as follows: $T_{0}^{layer1}=T_{0}^{layer3}=0^{\circ }$, $T_{0}^{layer2}=-45^{\circ }$, $T_{1}^{layer1}=T_{1}^{layer3}=45^{\circ }$, $T_{1}^{layer2}=-60^{\circ }$. The lamination of the plate is 90<0,45> for layer 1, 90<−45,−60> for layer 2 and 90<0,45> for layer 3. The stacking sequence is presented in Figure 5.

As for the previous case, the Abaqus reference solution contains 80 elements along each side and 12 elements along the thickness. For the CUF results, a 10×10 mesh is considered. Table 6 shows the first five natural frequencies for a/h=100, together with the results presented in Viglietti et al. [27].

In this case, the best approximation is given by the LM2 and LM4 theories. The LM2 and LM4 models both have a maximum percentage error as high as 0.4% corresponding to the third frequency. In addition, classical and low-order theories provide good results since a thin plate is considered. For this reason, transverse stresses do not play an important role. For example, the maximum error given by CLT is 2.1% for the fifth frequency. The case for a/h=10 is presented in Table 7.

Here, the CLT model shows that the inversion of the third and fourth modes can be observed by the corresponding values of the frequencies that are not in ascending order as the mode number increases. In comparison with the monolayer plate, in this case, the mode inversions of the CLT model can be seen for higher side-to-thickness ratios and lower frequencies. For the third mode, CLT shows a percentage error of 96.0%, while the best approximation is given by LM4, which has a percentage error of 0.17% for the same mode. Table 8 shows the first five frequencies for a/h=5.

In this case, lower-order theories have an evident loss of accuracy. The CLT model can predict only the first two modes. In addition, the FSDT and ED2 models show non-negligible errors, which become bigger with the increase in frequency. On the other hand, mixed models are able to correctly predict the dynamic behavior of the plate for both low and high frequencies.

### 3.3. Multilayer Plate with Central Hole

**Figure 6:**
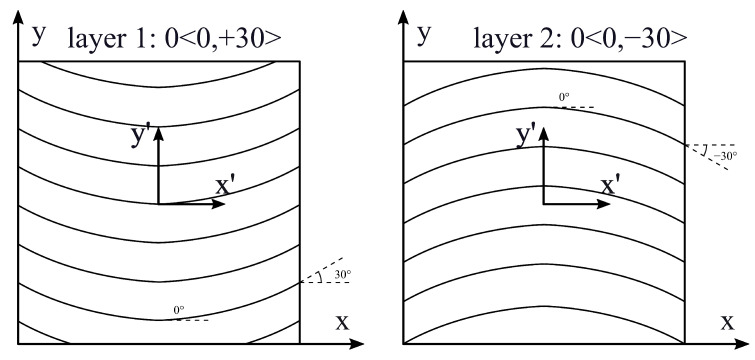
Stacking sequence; case 3.

Case 3 is taken from Hachemi et al. [9] and corresponds to a multilayer clamped plate that presents a circular cut-out. The center of the cut-out is placed at the plate center (a/2,b/2), and its radius is r=0.2 m. It is assumed that the fiber angle is a parabolic function of $x^{\prime }$, which means that α is parallel to $x^{\prime }$ ($\Phi =0^{\circ }$). As in the previous case, the $x^{\prime }$ and $y^{\prime }$ axes are parallel, respectively, to *x* and *y*, and their origin is placed at the center of the plate. The angle variational law is defined in Equation (12), considering d=a/2. The plate is composed of two layers that have the same thicknesses. The values of $T_{0}$ and $T_{1}$ are set for each layer as follows: $T_{0}^{layer1}=T_{0}^{layer2}=0^{\circ }$, $T_{1}^{layer1}=30^{\circ }$, $T_{1}^{layer2}=-30^{\circ }$. The stacking sequence is 0<0,±30>; see Figure 6.

In this case, the Abaqus reference solution is made of 73728 elements: 4608 elements are defined on the plate plane, and 16 elements are defined along the thickness. For the CUF results, 128 elements are used on the plate plane. The natural frequencies are expressed in the following dimensionless form:

(39) $$\bar{\omega }=\left(\omega a^{2}\right)\sqrt{\rho h/D_{0}}\;,$$

(40) $$D_{0}=E_{2}h^{3}/12\left(1-\nu_{LT}\nu_{TL}\right)\;,$$

where ω is the natural frequency, while $D_{0}$ represents a reference bending stiffness. Table 9 presents the first five non-dimensional frequencies for a/h=100.

It is possible to observe that the theories show a good approximation of the reference results. In addition, the percentage errors of FSDT and CLT are less than 2%. Mixed theories match the Abaqus results. Table 10 shows the results for a/h=10 in order to compare the Abaqus reference solution with the one presented in Hachemi et al. [9] and the solutions obtained with CUF.

**Figure 7:**
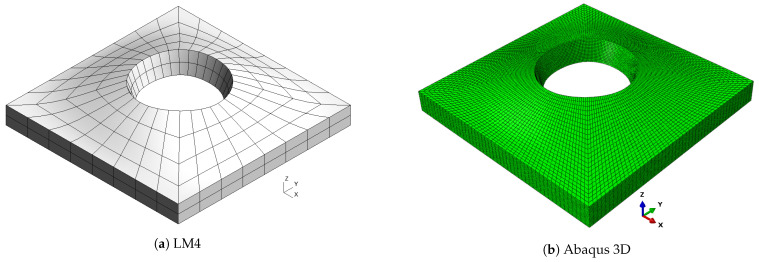
Mode 1, a/h=10; case 3.

**Figure 8:**
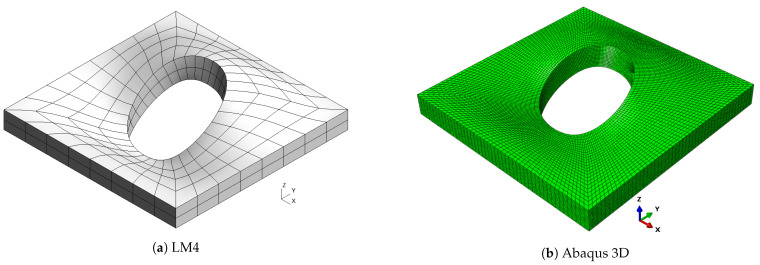
Mode 2, a/h=10; case 3.

**Figure 9:**
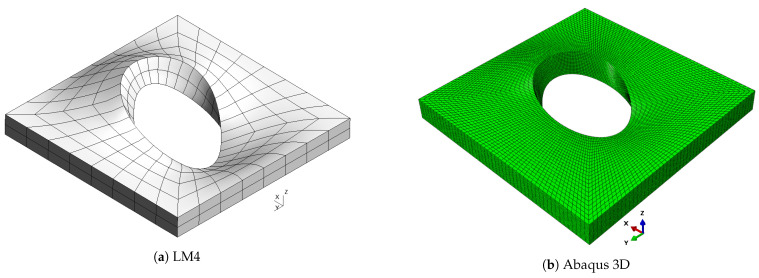
Mode 3, a/h=10; case 3.

**Figure 10:**
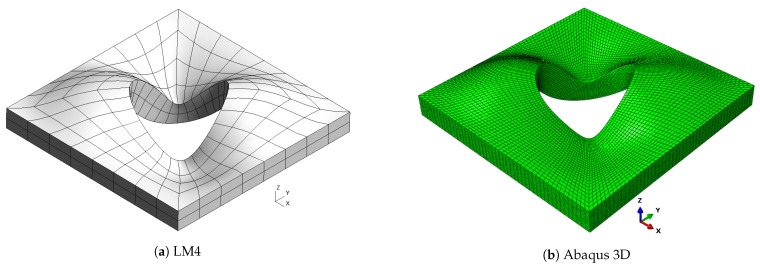
Mode 4, a/h=10; case 3.

**Figure 11:**
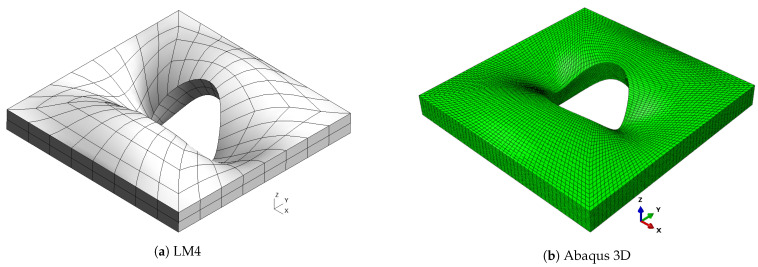
Mode 5, a/h=10; case 3.

As already observed in previous cases, classical theories and, in general, low-order ones are not able to provide an accurate approximation of natural frequencies, because of the low side-to-thickness ratio value. It is also possible that this generates the inversion of modes four and five for the CLT case. On the other hand, the best approximation is given by mixed theories, which are closer to the Abaqus solution for high frequencies. The shapes of the modes are presented in Figure 7, Figure 8, Figure 9, Figure 10 and Figure 11 for a/h=10. The modal shapes obtained with the LM4 model are compared with those of Abaqus 3D.

The first mode shows a simple bending of the plate on the xy plane with a single half-wave along each in-plane direction. The second and the third modes show two half-waves in the *y* and *x* directions, respectively. Mode number four shows three half-waves along the plate diagonally between the *x* and *y* axes. The fifth mode shows three half-waves along the *y* direction. Finally, Table 11 shows the frequencies for a/h=5.

Since a thick plate is considered, the effect of transverse stresses is not negligible, which causes the classical and lower-order theories to be inaccurate. This can be observed for CLT, which is not able to predict the fourth and fifth modes and has an error as high as 69.4% for the third mode. Considering the FSDT, ED4 and LD4 models, this error can be reduced to 4.4%, 0.5% and 0.1%, respectively.

## 4. Conclusions

In this paper, a new framework for the dynamic analysis of VAT structures is presented. RMVT is developed within CUF in order to obtain a new family of 2D models for the free-vibration analysis of VAT plates. The results are obtained via either RMVT or PVD and are compared in order to show the effective capabilities of the proposed method in the prediction of VAT plates’ natural frequencies. The Abaqus 3D reference solutions and results from Refs. [9,27] are also included to further validate the models proposed in this article. Linear and parabolic laws are both considered in order to describe the in-plane path of fiber variation. The possibility to use a polynomial order defined a priori through CUF and the introduction of the transverse stress field as a primary variable of the problem through RMVT both help to obtain a valid approach for the prediction of VAT dynamic behavior. After the results analysis, the following remarks can be made:

- Classical theories (FSDT and CLT) provide the best trade-off between accuracy and computational costs for thin plates (a/h=100), whereas they are not able to correctly predict the behavior of thicker plates (a/h=10 and 5), specially at high frequencies. The loss of accuracy is more evident for CLT results, since this theory does not consider transverse shear stresses, which become important in thick plates. This error is particularly evident in the second- and third-order theories, where the inversion of modes can be observed.
- The PVD results show monotonic convergence to the reference solution: the lower the DOF number, the higher the frequency value. For a given mode, frequency values decrease when higher-order models are employed, and they move closer to the reference solution.
- In all the cases, layer-wise mixed theories yield the best match of the reference 3D solution, independently from the plate geometry or fiber variational law. This is justified by the fact that RMVT considers both displacements and transverse stresses as primary variables, assuring a better approximation of the transverse stresses field into the problem domain, and improving the overall solution accuracy.
- For a given expansion order, models based on RMVT are more computationally expensive than PVD models. For this reason, the use of LW mixed models is advantageous in the cases where a more precise representation of the through-the-thickness behavior is needed, as in the case of higher frequencies or for thick plates, whereas low-order ESL and classical models are accurate for lower frequencies and thin plates.

In conclusion, the application of RMVT within CUF has demonstrated significant potential for improving the accuracy and efficiency of modeling VAT plates for free-vibration analyses. The promising results suggest, as future perspectives, the extension to buckling and failure analyses where an accurate and efficient modeling of VAT structures under various loading and operational conditions is required. 

## Figures and Tables

**Table 1:** Material properties.

Case	$E_{L}$ (GPa)	$E_{T}$ (GPa)	$G_{\mathrm{LT}}=G_{\mathrm{TT}}$ (GPa)	$\nu_{\mathrm{LT}}=\nu_{\mathrm{TT}}$
1	50.0	10.0	5.0	0.25
2	173.0	7.2	3.8	0.29
3	138.0	9.0	7.1	0.30

**Table 2:** Degrees of freedom; case 1.

Model	DOF
Abaqus 3D	997,515
3LM4	34,398
2LM2	13,230
3LD4	17,199
2LD2	6615
ED4	6615
ED2	3969
FSDT	2646
CLT	2646

**Table 3:** Natural frequencies (Hz), a/h=100; case 1.

	Mode				
	1	2	3	4	5
Abaqus 3D	7.397	16.354	37.158	48.025	63.349
3LM4	7.399	16.334	37.164	47.988	63.310
2LM2	7.398	16.333	37.162	47.986	63.309
3LD4	7.400	16.362	37.179	48.053	63.378
2LD2	7.400	16.362	37.179	48.054	63.379
ED4	7.400	16.362	37.179	48.053	63.378
ED2	7.401	16.368	37.186	48.069	63.399
FSDT	7.398	16.363	37.171	48.054	63.388
CLT	7.403	16.414	37.213	48.175	63.537

**Table 4:** Natural frequencies (Hz), a/h=10; case 1.

	Mode				
	1	2	3	4	5
Abaqus 3D	72.229	151.762	338.517	389.336	431.011
3LM4	72.244	151.751	338.577	389.554	431.004
2LM2	72.233	151.705	338.432	389.546	430.824
3LD4	72.250	151.796	338.625	389.587	431.151
2LD2	72.269	151.906	338.939	389.589	431.577
ED4	72.253	151.810	338.669	389.588	431.207
ED2	72.466	153.069	342.179	389.592	435.990
FSDT	72.437	153.021	342.036	389.510	435.853
CLT	73.825	163.064	365.813	389.510	472.565

**Table 5:** Natural frequencies (Hz), a/h=5; case 1.

	Mode				
	1	2	3	4	5
Abaqus 3D	136.723	264.080	389.391	556.394	704.284
3LM4	136.742	264.077	389.557	556.404	704.295
2LM2	136.667	263.747	389.550	555.332	703.121
3LD4	136.755	264.119	389.638	556.511	704.442
2LD2	136.875	264.684	389.643	558.145	706.381
ED4	136.774	264.224	389.640	556.855	704.832
ED2	138.015	269.553	389.651	570.563	721.354
FSDT	137.947	269.463	389.510	570.329	721.159
CLT	146.479	319.929	389.510	696.687	895.089

**Table 6:** Natural frequencies (Hz), a/h=100; case 2.

	Mode				
	1	2	3	4	5
Abaqus 3D	92.18	130.68	194.96	237.56	274.60
Ref. [27]	92.90	132.28	198.97	240.46	278.75
LM4	92.35	131.01	195.77	238.25	275.60
LM2	92.34	130.99	195.74	238.23	275.58
LD4	92.36	131.03	195.81	238.30	275.67
LD2	92.36	131.04	195.84	238.31	275.69
ED4	92.37	131.06	195.88	238.32	275.72
ED2	92.49	131.23	196.16	238.97	276.48
FSDT	92.38	131.01	195.75	238.74	276.20
CLT	93.04	131.85	197.00	242.48	280.40

**Table 7:** Natural frequencies (Hz), a/h=10; case 2.

	Mode				
	1	2	3	4	5
Abaqus 3D	606.67	896.70	1208.24	1313.26	1458.25
Ref. [27]	609.79	903.63	1216.00	1328.41	1469.33
LM4	606.90	897.26	1208.80	1314.85	1459.23
LM2	606.33	896.52	1206.86	1313.56	1457.30
LD4	607.22	897.73	1209.64	1315.80	1460.16
LD2	608.65	901.20	1213.06	1322.93	1465.20
ED4	609.84	905.18	1214.60	1331.82	1469.17
ED2	633.68	941.96	1272.39	1396.16	1540.10
FSDT	632.82	940.46	1271.42	1393.96	1538.74
CLT	921.28	1287.71	2368.22	1885.61	2699.22

**Table 8:** Natural frequencies (Hz), a/h=5; case 2.

	Mode				
	1	2	3	4	5
Abaqus 3D	794.730	1201.916	1439.956	1701.328	1810.250
LM4	794.760	1202.101	1440.092	1701.788	1811.113
LM2	792.734	1199.331	1433.897	1696.266	1805.942
LD4	795.213	1202.777	1441.080	1702.986	1812.317
LD2	799.063	1209.706	1448.714	1713.982	1820.716
ED4	802.019	1216.744	1450.930	1723.900	1825.405
ED2	845.154	1294.481	1523.246	1847.193	1930.364
FSDT	844.048	1292.846	1522.478	1845.945	1928.631
CLT	1790.121	2411.198	-	-	-

**Table 9:** Non-dimensional frequencies $\bar{\omega }$, a/h=100; case 3.

	Mode				
	1	2	3	4	5
Abaqus 3D	87.079	106.407	147.559	184.034	197.096
LM4	87.281	106.622	147.070	184.554	197.522
LM2	87.259	106.593	147.045	184.500	197.489
LD4	87.327	106.704	147.911	184.789	197.969
LD2	87.336	106.719	147.952	184.821	198.022
ED4	87.331	106.708	147.921	184.798	197.984
ED2	87.364	106.768	148.169	184.931	198.228
FSDT	87.184	106.538	148.047	184.525	198.029
CLT	87.387	106.942	150.080	185.420	199.725

**Table 10:** Non-dimensional frequencies $\bar{\omega }$, a/h=10; case 3.

	Mode				
	1	2	3	4	5
Abaqus 3D	72.645	86.745	104.279	136.366	140.278
Ref. [9]	72.432	86.626	103.910	135.828	139.747
LM4	72.699	86.830	104.307	136.467	140.408
LM2	72.573	86.700	104.051	136.137	140.143
LD4	72.744	86.888	104.376	136.558	140.516
LD2	73.107	87.263	105.144	137.567	141.231
ED4	72.868	86.990	104.630	136.851	140.725
ED2	73.977	88.609	107.143	140.522	143.556
FSDT	74.075	88.782	107.645	141.221	143.885
CLT	84.751	104.166	143.133	190.321	174.656

**Table 11:** Non-dimensional frequencies $\bar{\omega }$, a/h=5; case 3.

	Mode				
	1	2	3	4	5
Abaqus 3D	54.333	64.456	70.572	90.875	98.086
LM4	54.326	64.456	70.541	90.866	98.098
LM2	54.038	64.201	70.036	90.292	97.612
LD4	54.388	64.514	70.619	90.956	98.191
LD2	54.875	64.963	71.421	91.868	98.955
ED4	54.554	64.623	70.913	91.224	98.408
ED2	56.062	66.756	73.181	94.442	101.535
FSDT	56.253	66.985	73.702	95.219	102.017
CLT	76.928	95.975	119.513	-	-

## Data Availability

Not applicable.

## References

[1] Dirk H.J.L., Ward C., Potter K.D. (2012). The engineering aspects of automated prepreg layup: History, present and future. Compos. Part B Eng..

[2] Zhuo P., Li S., Ashcroft I.A., Jones A.I. (2021). Material extrusion additive manufacturing of continuous fiber reinforced polymer matrix composites: A review and outlook. Compos. Part B Eng..

[3] Brooks T.R., Martins J.R., Kennedy G.J. (2019). High-fidelity aerostructural optimization of tow-steered composite wings. J. Fluids Struct..

[4] Grenoble R.W., Nguyen T., McKenney M.J., Przekop A., Juarez P.D., Gregory E.D., Jegley D.C. Fabrication of a composite tow-steered structure for air-launch vehicle applications. Proceedings of the AIAA/ASCE/AHS/ASC Structures, Structural Dynamics, and Materials Conference.

[5] Hyer M.W., Charette R.F. (1990). Innovative Design of Composite Structures: The Use of Curvilinear Fiber Format in Composite Structure Design.

[6] Hyer M.W., Lee H.H. (1991). Innovative design of composite structures: The use of curvilinear fiber format to improve buckling resistance of composite plates with central circular holes. Compos. Struct..

[7] Akhavan H., Ribeiro P. (2011). Natural modes of vibration of variable stiffness composite laminates with curvilinear fibers. Compos. Struct..

[8] Ribeiro P., Akhavan H. (2012). Non-linear vibrations of variable stiffness composite laminated plates. Compos. Struct..

[9] Hachemi M., Hamza-Cherif S.M., Houmat A. (2020). Free vibration analysis of variable stiffness composite laminate plate with circular cutout. Aust. J. Mech. Eng..

[10] Zhao W., Kapania R.K. (2019). Prestressed vibration of stiffened variable-angle tow laminated plates. AIAA J..

[11] Honda S., Narita Y. (2012). Natural frequencies and vibration modes of laminated composite plates reinforced with arbitrary curvilinear fiber shape paths. J. Sound Vib..

[12] Rodrigues J.D., Ribeiro P., Akhavan H. Experimental and finite element modal analysis of variable stiffness composite laminated plates. Proceedings of the 11th Biennial International Conference on Vibration Problems (ICOVP-2013).

[13] Stodieck O., Cooper J.E., Weaver P.M., Kealy P. (2013). Improved aeroelastic tailoring using tow-steered composites. Compos. Struct..

[14] Abdalla M.M., Setoodeh S., Gürdal Z. (2007). Design of variable stiffness composite panels for maximum fundamental frequency using lamination parameters. Compos. Struct..

[15] Blom A.W., Setoodeh S., Hol J., Gürdal Z. (2008). Design of variable-stiffness conical shells for maximum fundamental eigenfrequency. Comput. Struct..

[16] Carvalho J., Sohouli A., Suleman A. (2022). Fundamental Frequency Optimization of Variable Angle Tow Laminates with Embedded Gap Defects. J. Compos. Sci..

[17] Montemurro M., Catapano A. (2017). On the effective integration of manufacturability constraints within the multi-scale methodology for designing variable angle-tow laminates. Compos. Struct..

[18] Catapano A., Montemurro M., Balcou J.-A., Panettieri E. (2019). Rapid prototyping of variable angle-tow composites. Aerotec. Missili Spaz..

[19] Montemurro M., Catapano A. (2019). A general B-Spline surfaces theoretical framework for optimisation of variable angle-tow laminates. Compos. Struct..

[20] Fiordilino G.A., Izzi M.I., Montemurro M. (2021). A general isogeometric polar approach for the optimisation of variable stiffness composites: Application to eigenvalue buckling problems. Mech. Mater..

[21] Carrera E. (2002). Theories and finite elements for multilayered, anisotropic, composite plates and shells. Arch. Comput. Meth. Eng..

[22] Carrera E. (2003). Theories and finite elements for multilayered plates and shells: A unified compact formulation with numerical assessment and benchmarking. Arch. Comput. Meth. Eng..

[23] Carrera E., Giunta G., Brischetto S. (2007). Hierarchical closed form solutions for plates bent by localized transverse loadings. J. Zhejiang Univ. Sci. A.

[24] Carrera E., Giunta G. (2008). Hierarchical models for failure analysis of plates bent by distributed and localized transverse loadings. J. Zhejiang Univ. Sci. A.

[25] Giunta G., Catapano A., Belouettar S. (2013). Failure indentation analysis of composite sandwich plates via hierarchical models. J. Sandw. Struct. Mater..

[26] Giunta G., Biscani F., Belouettar S., Ferreira A.J.M., Carrera E. (2013). Free vibration analysis of composite beams via refined theories. Compos. Part B Eng..

[27] Viglietti A., Zappino E., Carrera E. (2019). Analysis of variable angle tow composites structures using variable kinematic models. Compos. Part B Eng..

[28] Fallahi N., Viglietti A., Carrera E., Pagani A., Zappino E. (2020). Effect of fiber orientation path on the buckling, free vibration, and static analyses of variable angle tow panels. Facta Univ. Ser. Mech. Eng..

[29] Sánchez-Majano A.R., Azzara R., Pagani A., Carrera E. (2021). Accurate Stress Analysis of Variable Angle Tow Shells by High-Order Equivalent-Single-Layer and Layer-Wise Finite Element Models. Materials.

[30] Pagani A., Sánchez-Majano A.R. (2022). Influence of fiber misalignments on buckling performance of variable stiffness composites using layerwise models and random fields. Mech. Adv. Mater. Struct..

[31] Pagani A., Sánchez-Majano A.R. (2021). Stochastic stress analysis and failure onset of variable angle tow laminates affected by spatial fiber variations. Compos. Part C Open Access.

[32] Sánchez-Majano A.R., Pagani A., Petrolo M., Zhang C. (2021). Buckling sensitivity of tow-steered plates subjected to multiscale defects by high-order finite elements and polynomial chaos expansion. Materials.

[33] Vescovini R., Dozio L. (2016). A variable-kinematic model for variable stiffness plates: Vibration and buckling analysis. Compos. Struct..

[34] Demasi L., Biagini G., Vannucci F., Santarpia E., Cavallaro R. (2017). Equivalent Single Layer, Zig-Zag, and Layer Wise theories for variable angle tow composites based on the Generalized Unified Formulation. Compos. Struct..

[35] Carrera E., Demasi L. (2002). Classical and advanced multilayered plate elements based upon PVD and RMVT. Part 1: Derivation of finite element matrices. Int. J. Numer. Methods Eng..

[36] Carrera E., Demasi L. (2002). Classical and advanced multilayered plate elements based upon PVD and RMVT. Part 2: Numerical implementations. Int. J. Numer. Methods Eng..

[37] Babaei M., Kiarasi F., Tehrani M.S., Hamzei A., Mohtarami E., Asemi K. (2022). Three dimensional free vibration analysis of functionally graded graphene reinforced composite laminated cylindrical panel. Proc. Inst. Mech. Eng. Part L J. Mater. Des. Appl..

[38] Reddy J.N. (2003). Mechanics of Laminated Composite Plates and Shells: Theory and Analysis.

[39] Gürdal Z., Tatting B.F., Wu C.K. (2008). Variable stiffness composite panels: Effects of stiffness variation on the in-plane and buckling response. Compos. Part A Appl. Sci. Manuf..

[40] Honda S., Oonishi Y., Narita Y., Sasaki K. (2008). Vibration analysis of composite rectangular plates reinforced along curved lines. J. Syst. Des. Dyn..

[41] Carrera E. (2004). On the use of the Murakami’s zig-zag function in the modeling of layered plates and shells. Comput. Struct..

[42] Bathe K.-J. (2006). Finite Element Procedures.

